# Establishment and validation of a 28-day mortality prediction model based on the lactate dehydrogenase/albumin ratio in patients with severe pneumonia

**DOI:** 10.3389/fmed.2025.1696945

**Published:** 2026-01-21

**Authors:** Kai Ma, Li Zhou, Hong Luo

**Affiliations:** 1 Department of Pulmonary and Critical Care Medicine, The Second Xiangya Hospital, Central South University, Changsha, China; 2 Research Unit of Respiratory Disease, Central South University, Changsha, China; 3 Clinical Medical Research Center for Pulmonary and Critical Care Medicine in Hunan Province, Changsha, China; 4 Diagnosis and Treatment Center of Respiratory Disease in Hunan Province, Changsha, China

**Keywords:** severe pneumonia, lactate dehydrogenase to albumin ratio, mortality prediction, sepsis, machine learning

## Abstract

**Background:**

Severe pneumonia (SP) is a common and often fatal disease. Traditional mortality risk assessments rely on complex scoring systems and lack simple, effective biomarkers. This study aims to explore the potential value of the lactate dehydrogenase to albumin ratio (LAR) in predicting 28-day mortality in patients with severe pneumonia and to develop a predictive model using machine learning techniques.

**Methods:**

This retrospective cohort study included patients with severe pneumonia admitted to the Second Xiangya Hospital of Central South University, from January 2020 to May 2025. Clinical data, laboratory indicators, and LAR values were collected. Cox regression analysis, the Boruta feature selection algorithm, and various machine learning models were employed for analysis. The primary outcome was 28-day survival status, and the relationship between LAR and mortality risk was evaluated, leading to the development of a prediction model based on LAR.

**Results:**

Among the 491 patients, LAR was significantly associated with 28-day mortality risk and was identified as an independent risk factor for death in severe pneumonia. LAR demonstrated a high area under the curve (AUC) in predicting mortality, exhibiting a significant nonlinear relationship with 28-day mortality risk. Among several machine learning models constructed using key variables selected by the Boruta algorithm, the random forest (RF) model exhibited the best predictive performance. Furthermore, Shapley additive explanations (SHAP) value analysis confirmed the dominant role of LAR in the RF model.

**Conclusion:**

LAR is an effective biomarker with significant clinical value in predicting 28-day mortality in patients with severe pneumonia. The LAR-based prediction model enhances the accuracy of mortality risk assessment, especially in non-septic patients. Combined with machine learning techniques, LAR offers a novel tool for early clinical risk evaluation and holds promising potential for clinical application.

## 1 Introduction

Severe pneumonia is one of the most common and serious infectious diseases in clinical practice, particularly in elderly patients and those with underlying conditions, where the incidence is notably higher, posing a significant threat to patient survival. It accounts for approximately 10–20% of hospitalized pneumonia cases and over 20% of infectious disease admissions to intensive care units (ICU) (1, 2). Despite significant advancements in antimicrobial therapy, mechanical ventilation technologies, and intensive care support methods in recent years, the overall diagnosis and treatment level for severe pneumonia has notably improved. However, its mortality rate remains alarmingly high, with some studies reporting a mortality rate as high as 50% (3, 4). Currently, the assessment of mortality risk in severe pneumonia patients largely relies on various scoring systems, such as the Acute Physiology and Chronic Health Evaluation II (APACHE II) and the Sequential Organ Failure Assessment (SOFA). While these scoring systems can reflect the severity of a patient’s condition to some extent, their application is often complex, with cumbersome calculations that limit their widespread use and real-time implementation in clinical practice. Therefore, the search for a simple, objective, and easily applicable biomarker with strong predictive performance to establish a practical mortality risk prediction model has become one of the key areas of focus in current severe pneumonia research.

Lactate dehydrogenase (LDH) is a cytoplasmic enzyme widely distributed across various tissues and cells in the human body, primarily involved in the glycolytic process of glucose metabolism (5). During cell injury, tissue ischemia, or systemic inflammatory responses, LDH can be released from cells into the bloodstream, reflecting the extent of tissue damage and the inflammatory state. Elevated serum LDH levels often indicate cellular structural damage and systemic inflammatory activation, and it is especially sensitive in infectious diseases (6). In patients with severe pneumonia, the glycolytic pathway in alveolar epithelial cells is activated, leading to the production of significant amounts of ATP and lactate (7). As one of the key enzymes involved in this metabolic process, LDH plays a crucial role. Studies have shown that LDH levels are significantly associated with disease severity and mortality risk in patients with novel coronavirus pneumonia (COVID-19) (8), sepsis (9), and severe pneumonia following kidney transplantation (10). Albumin, the major plasma protein synthesized by the liver, plays a significant role in maintaining plasma colloid osmotic pressure, transporting endogenous and exogenous substances, and other physiological functions (11). Its levels are regulated by various factors, including nutritional status, liver function, and inflammation. Under inflammatory conditions, due to increased permeability of capillaries and accelerated catabolism of albumin, the concentration of plasma albumin usually drops significantly (12), serving as an indicator of overall nutrition and a direct measure of the inflammatory response (13). Hypoalbuminemia has been confirmed to be closely associated with poor prognosis in various diseases, such as inflammatory diseases, sepsis, and acute respiratory distress syndrome (ARDS) (14, 15). A prospective observational study further revealed that in sepsis patients, admission albumin levels were negatively correlated with 30-day mortality. For every 1 g/dL decrease in albumin, the mortality risk increased approximately threefold (16).

The lactate dehydrogenase to albumin ratio (LAR) is a novel composite biomarker that has gained widespread attention in recent years for its role in assessing inflammatory responses and tissue damage. This ratio can, to some extent, comprehensively reflect the extent of cellular damage and the nutritional-inflammatory status of the body. Elevated LAR levels have been significantly associated with increased mortality in sepsis patients (17, 18). Additionally, studies have confirmed that in COVID-19 patients, LAR serves as an independent predictor of mortality (19). However, the prognostic value of LAR in patients with severe pneumonia remains insufficiently studied, and its relationship with adverse outcomes in these patients remains unclear.

This study aims to retrospectively analyze the clinical data of hospitalized severe pneumonia patients to explore the association between LAR levels and 28-day mortality. By utilizing machine learning techniques and integrating clinical characteristics and laboratory markers, we aim to construct a mortality risk prediction model based on LAR. The goal of this study is to develop a simple and practical early risk identification tool, providing support for personalized treatment and optimizing resource allocation in clinical practice.

## 2 Materials and methods

### 2.1 Study subjects

This study is a retrospective cohort study, in which we collected data from patients with severe pneumonia admitted to the Second Xiangya Second Hospital of Central South University between January 2020 and May 2025.

Diagnostic Criteria: According to the guidelines of the Infectious Diseases Society of America (IDSA) and the American Thoracic Society (ATS) (20), patients with severe pneumonia must meet one major criterion or at least three minor criteria. The major criteria include: (1) the need for mechanical ventilation via endotracheal intubation, and (2) the requirement for vasopressor therapy despite adequate fluid resuscitation for infectious shock. The minor criteria include: (1) respiratory rate ≥ 30 breaths per minute, (2) the ratio of arterial oxygen pressure to inspired oxygen fraction (PaO2/FiO2) ≤ 250, (3) multilobar infiltrates, (4) altered consciousness or disorientation, (5) blood urea nitrogen ≥ 7.14 mmol/L, and (6) systolic blood pressure < 90 mmHg, necessitating aggressive fluid resuscitation.

Inclusion Criteria: (1) Patients who meet the diagnostic criteria for severe pneumonia; (2) Hospital admission duration > 24 h; (3) Age > 18 years.

Exclusion Criteria: (1) Patients with malignancies; (2) Hematological disorders; (3) Acute or chronic viral hepatitis; (4) Patients with a known history of renal dysfunction and those undergoing dialysis treatment; (5) Post-transplant immunosuppressive state and patients with immune disorders (Rheumatic diseases); (6) Pregnant women.

This study adheres to ethical standards in medical research and has received approval from the hospital’s ethics committee (Approval No: 2021(160)).

### 2.2 Data collection

Data collected from patients within 24 h of admission, including basic demographic information (gender, age), vital signs at the time of admission (temperature, heart rate, respiratory rate, systolic blood pressure (SBP), diastolic blood pressure(DBP), mean arterial pressure (MAP)), medical history (hypertension, diabetes, coronary artery disease(CHD), chronic obstructive pulmonary disease(COPD)), comorbidities (such as COVID-19 and sepsis), and disease severity scores (CURB-65, SOFA, qSOFA). Laboratory markers included: white blood cell count (WBC), neutrophils, lymphocytes, monocytes, red blood cell count (RBC), hemoglobin (Hb), platelets count (PLT), albumin, lactate dehydrogenase (LDH), total bilirubin, alanine aminotransferase (ALT), aspartate aminotransferase (AST), blood urea nitrogen (BUN), creatinine, uric acid, C-reactive protein (CRP), procalcitonin (PCT), sodium (Na), potassium (K), prothrombin time (PT), international normalized ratio (INR), bicarbonate (HCO₃^−^), and anion gap (AG). Interventions included the use of vasopressors and invasive mechanical ventilation (IMV). The primary outcome measure was 28-day mortality.

The lactate dehydrogenase to albumin ratio (LAR) was calculated as follows: LAR = Lactate dehydrogenase (U/L)/Albumin (g/L).

Mean arterial pressure (MAP) = (systolic blood pressure + 2 × diastolic blood pressure)/3.

### 2.3 Statistical methods

Data processing and analysis were performed using SPSS and R 4.1.1 software. For continuous variables, normality and homogeneity of variance tests were conducted. Normally distributed data were expressed as mean ± standard deviation (x ± s), and comparisons between groups were made using the t-test. Non-normally distributed data were expressed as median (interquartile range) [M (Q1, Q3)], and comparisons between groups were made using the Mann–Whitney U test. Categorical data were presented as percentages, and comparisons were performed using the chi-square test. To control for false positives resulting from multiple comparisons, FDR correction was applied to the results of intergroup variable comparisons. *p*-value of <0.05 was considered statistically significant.

### 2.4 Regression analysis

Univariate and multivariate Cox proportional hazards regression models were used to assess potential risk factors influencing the 28-day mortality in severe pneumonia patients. Before performing the multivariate Cox regression analysis, multicollinearity among variables was assessed using the Variance Inflation Factor (VIF). Based on the optimal cutoff value of LAR, patients were divided into groups, and Kaplan–Meier survival curves were plotted to evaluate the impact of LAR on survival outcomes.

### 2.5 Boruta method and restricted cubic spline (RCS)

The Boruta package in R was used for feature selection of all candidate variables. In the resulting boxplot, green represents important features, defined as those with a Z-value greater than the highest Z-value of shadow features and exceeding the significance threshold. Blue represents intermediate state variables, which are defined as variables temporarily retained during the iterative feature selection process, neither meeting the significance threshold nor being definitively excluded. Red represents unimportant variables, defined as those with a Z-value smaller than the highest Z-value of shadow features. Only variables classified as “important” were included in the final model.

To investigate whether there is a non-linear relationship between LAR and 28-day mortality risk, a restricted cubic spline (RCS) model with four knots was used for fitting analysis.

### 2.6 Stratification and interaction analysis

Stratified analysis was first performed to explore the relationship between the lactate dehydrogenase to albumin ratio (LAR) and 28-day mortality across different subgroups. If significant differences or trends were observed in a specific subgroup, interaction analysis was further conducted to examine whether there was a statistical interaction effect between LAR and the subgroup variable. The specific stratifications included age (< 65 years vs. ≥ 65 years), gender (female vs. male), COVID-19 (yes vs. no), and sepsis (yes vs. no).

### 2.7 Construction and evaluation of the predictive model

Before model construction, samples with missing values were excluded. Categorical variables were incorporated into the analysis after one-hot encoding, and continuous variables were standardized. After excluding treatment-related variables, the Boruta method was used to select the remaining variables (*p* = 0.05, maximum trees = 1,000, maximum iterations = 500) to identify predictive factors associated with 28-day mortality risk. After removing evaluation-related categorical variables, the remaining variables were incorporated into the final model. The dataset was split into training and validation sets in a 3:1 ratio (21), with stratified sampling employed to ensure consistent 28-day mortality rates between the two groups. Nine machine learning models were developed in the training set: Logistic Regression, Decision Tree (DT), Elastic Net (Enet), K-Nearest Neighbors (KNN), Lightgbm (Light gradient boosting machine), Random Forest (RF), Xgboost, Support Vector Machine (SVM), and Multilayer Perceptron (MLP). Hyperparameter optimization was performed using a Bayesian optimization approach based on the average AUC from 10-fold cross-validation within the training set, ensuring the reliability and generalizability of the tuning process. To prevent overfitting and enhance the robustness and generalizability of the models, appropriate regularization methods were applied, including L1/L2 penalties (e.g., ENet and Logistic Regression), structural complexity control (e.g., maximum depth, minimum leaf samples, and split thresholds in tree models), and the optimization of sampling ratios, learning rate, and weight decay parameters. The performance of all models in the validation set was assessed using several evaluation metrics, including the area under the receiver operating characteristic curve (AUC), calibration curve, decision curve analysis (DCA), sensitivity, specificity, and others. The performance of the best model was further analyzed using Shapley Additive Explanations (SHAP) to quantify and visualize the relative contributions of each feature variable to the model’s predictions, clarifying their impact on the final outcome.

## 3 Results

### 3.1 Baseline characteristics

A total of 491 patients with severe pneumonia were included in this study, with 345 survivors (84.6%) and 146 non-survivors (15.4%). Among the patients, 356 were male and 135 were female. A comparison of baseline characteristics between the mortality group and the survival group revealed significant differences in age, vital signs, laboratory markers, clinical scores, and comorbidities (Table 1). In terms of vital signs, the respiratory rate was significantly higher in the death group than in the survival group (both *p* < 0.05, adjusted *p* < 0.05). In laboratory tests, significant differences were observed between the two groups in LDH, LAR, creatinine, BUN, PCT, and AG (all *p* < 0.05, adjusted *p* < 0.05). Regarding clinical severity scores, the mortality group had significantly higher SOFA, qSOFA, and CURB-65 scores compared to the survival group (all *p* < 0.05, adjusted *p* < 0.05), indicating more severe disease in the former group. With regard to comorbidities, the proportion of patients with concurrent COVID-19 infection and sepsis was significantly higher in the mortality group than in the survival group (both *p* < 0.05, adjusted *p* < 0.05). Additionally, a higher proportion of patients in the mortality group received vasopressors and mechanical ventilation during treatment compared to those in the survival group (both *p* < 0.05, adjusted *p* < 0.05).

**Table 1:** Patient demographics and baseline characteristics.

Characteristics	Non-survivor (*N* = 146)	Survivor (*N* = 345)	*P*-value	FDR-adjusted *P*
Demographics				
Age (years), median (Q1-Q3)	74 (64–82)	69.00 (59–79)	< 0.001	0.004
Gender, Male, *n* (%)	114 (78.1)	242 (70.1)	0.072	0.151
Vital signs				
Temperature (°C)	36.8 (36.5–37.5)	36.7 (36.5–37)	0.033	0.091
Heart rate (bpm)	96 (85–110)	95 (81–110)	0.832	0.870
Respiratory rate (bpm)	23 (20–25)	21 (20–24)	0.015	0.044
DBP (mmHg)	70.5 ± 15.7	73.3 ± 14.0	0.054	0.235
SBP (mmHg)	128 (112–140)	132 (111–146)	0.149	0.260
MAP (mmHg)	91.17 (78–99)	92.67 (81.33–102.67)	0.103	0.206
Laboratory indicators				
WBC (×10^9^/L)	9.55 (6.54–14.25)	8.38 (6.33–12.41)	0.047	0.115
Neutrophil (×10^9^/L)	7.98 (5.49–12.68)	7.28 (5.14–10.68)	0.055	0.128
Lymphocyte (×10^9^/L)	0.66 (0.43–1.04)	0.67 (0.38–1.03)	0.461	0.569
Monocyte (×10^9^/L)	0.38 (0.20–0.62)	0.35 (0.21–0.54)	0.270	0.390
RBC (×10^9^/L)	3.68 (3.17–4.22)	3.63 (3.09–4.08)	0.268	0.390
Hb (g/L)	109.4 ± 25.5	108.1 ± 23.1	0.594	0.710
PLT (×10^9^/L)	183.5 (129–234)	177 (123–270)	0.717	0.773
Albumin (g/L)	28.75 (25.2–32.2)	29.3 (26.7–33.2)	0.035	0.091
LDH (U/L)	480.5 (337.9–638)	339 (262–473.8)	< 0.001	< 0.001
LAR	15.24 (11.76–26.13)	11.43 (8.76–16.26)	< 0.001	< 0.001
PT (s)	14.70 (1.39–77.90)	15.00 (1.21–79)	0.651	
INR	1.13 (1.05–1.21)	1.11 (1.04–1.23)	0.442	
Bilirubin (umol/L)	10.10 (6.80–14.40)	9.5 (6.8–14.4)	0.849	0.870
ALT (U/L)	26.15 (14.30–45.20)	23.90 (15.60–40.60)	0.586	0.703
AST (U/L)	36.55 (22.90–66.3)	34.20 (23.50–56.90)	0.247	0.384
BUN (mmol/L)	10.05 (6.70–16.90)	8.80 (5.80–13.40)	0.014	0.044
Creatinine (umol/L)	98.00 (63.00–170.00)	81.00 (61.20–122.00)	0.014	0.044
Uric acid (umol/L)	251.30 (179.00–415.00)	245.00 (156.00–353.00)	0.154	0.230
CRP (mg/l)	104.00 (49.50–183.47)	92.50 (42.4–174.00)	0.134	0.245
PCT (ng/ml)	0.92 (0.26–3.41)	0.50 (0.17–1.71)	< 0.001	0.003
Na (mmol/L)	138.80 (135.00–142.40)	138.00 (134.90–142.10)	0.386	0.507
K (mmol/L)	4.06 (3.50–4.40)	3.87 (3.50–4.34)	0.202	0.326
HCO3^−^ (mmol/L)	22.3 (18.5–26.2)	22.5 (20.20–25.20)	0.718	0.773
AG (mmol/L)	18.05 (15.9–21.00)	17.3 (15.6–19.5)	0.011	0.042
Medical scores, median (Q1–Q3)				
SOFA (score)	5 (3–8)	3 (2–5)	< 0.001	< 0.001
qSOFA (score)	1 (0–2)	1 (0–1)	0.006	0.024
CURB-65 (score)	2 (1–3)	2 (1–2)	< 0.001	0.002
Treatments, *n* (%)				
Vasopressors	116 (79.5)	75 (21.7)	< 0.001	< 0.001
MV	110 (75.3)	152 (44.1)	< 0.001	< 0.001
Comorbidity disease, *n* (%)				
COPD	19 (13.0)	58 (16.8)	0.342	0.499
CHD	44 (30.1)	89 (25.8)	0.320	0.510
Diabetes	48 (32.9)	114 (33.0)	0.971	0.972
Hypertension	63 (43.2)	182 (52.8)	0.061	0.143
COVID-19	82 (56.2)	108 (31.3)	< 0.001	< 0.001
Sepsis	68 (46.6)	98 (28.4)	< 0.001	< 0.001

SOFA, sequential organ failure assessment; qSOFA, quick sequential organ failure assessment; DBP, diastolic blood pressure; SBP, systolic blood pressure; MAP, mean arterial pressure; WBC, white blood cell count; RBC, red blood cell count; Hb, hemoglobin; LDH, lactate dehydrogenase; LAR, lactate dehydrogenase to albumin ratio; PLT, platelet counts; INR, international normalized ratio; PT, prothrombin time; ALT, alanine aminotransferase; AST, aspartate aminotransferase; BUN, blood urea nitrogen; AG, anion gap; CRP, C-reactive protein; PCT, procalcitonin; IMV, invasive mechanical ventilation; CHD, coronary heart disease.

### 3.2 ROC curve and survival analysis

**Figure 1:**
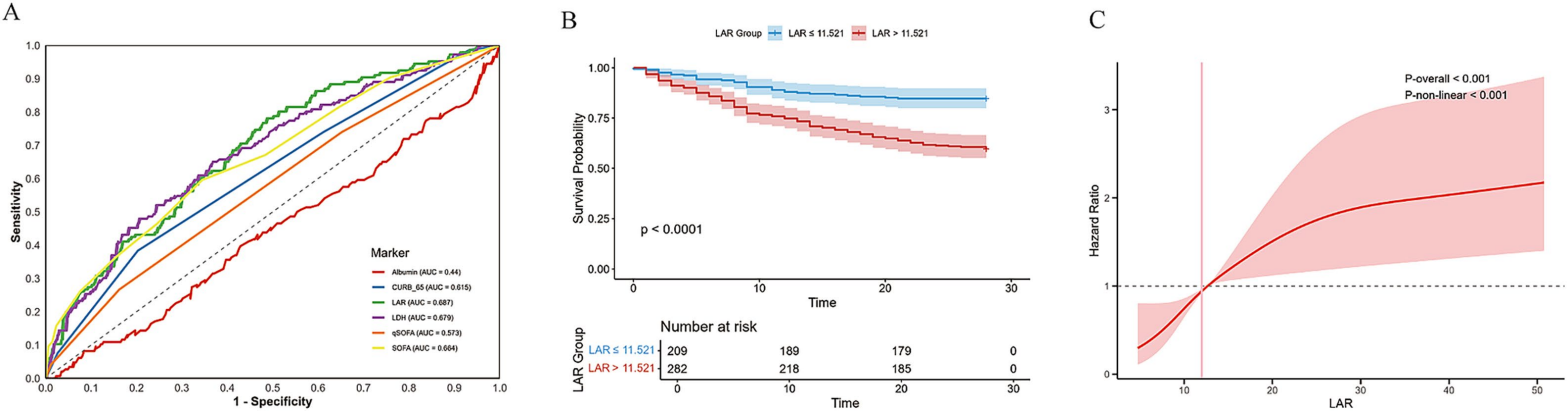
The relationship between LAR and the 28-day mortality rate of patients with severe pneumonia. **(A)** ROC curve for 28 day mortality. **(B)** 28-day KM survival curve. KM curve shows the 28-day survival rates of patients with severe pneumonia at different LAR levels. **(C)** RCS analysis of LAR and mortality in patients with severe pneumonia.

To evaluate the diagnostic performance of LAR in predicting 28-day mortality in severe pneumonia patients, a ROC curve was constructed (Figure 1A). The results showed that the area under the curve (AUC) for LAR was 0.687, which was superior to that of LDH (AUC = 0.679), albumin (AUC = 0.440), CURB-65 (AUC = 0.615), qSOFA (AUC = 0.573), and SOFA (AUC = 0.664), suggesting that LAR has a relatively high ability in predicting short-term mortality risk.

The optimal cutoff value for LAR to predict 28-day mortality was determined to be 11.521 using the Youden index. Based on this value, patients were divided into two groups: high LAR group (LAR > 11.521) and low LAR group (LAR ≤ 11.521), and Kaplan–Meier survival curves were plotted for further analysis (Figure 1B). The results showed that the 28-day survival rate was significantly higher in the low LAR group compared to the high LAR group (40.4%, 114/282 vs. 15.3%, 32/209), and the difference was statistically significant (Log-rank test: χ^2^ = 34.4, df = 1, *p* < 0.001). Additionally, the survival time of patients in the high LAR group was significantly shorter, suggesting that LAR may serve as a potential biomarker for poor prognosis.

### 3.3 Independent risk factors

Univariate Cox proportional hazards regression analysis was performed on all clinical variables, and the results (Table 2) revealed that Age, Temperature, DBP, Albumin, LDH, LAR, WBC, Neutrophil, PCT, ALT, BUN, Creatinine, Uric Acid, AG, SOFA, qSOFA, CURB-65, and comorbidities such as COVID-19 and Sepsis were significantly associated with the 28-day mortality outcome (all *p* < 0.05). Based on the multicollinearity test results (Supplementary Tables 4, 5), LDH and WBC (VIF > 10) were excluded, while the remaining variables (VIF < 5) were included in the multivariate Cox regression analysis. The results demonstrated that LAR, age, temperature, SOFA score, neutrophil count, albumin level, coexisting COVID-19, and sepsis were independent risk factors for 28-day mortality in patients with severe pneumonia (all *p* < 0.05), further supporting the clinical value of LAR as a key prognostic indicator.

**Table 2:** Univariable cox regression analysis and multivariable cox regression analysis for 28- day mortality.

Characteristics	Univariable cox regression			Multivariable cox regression		
	HR	95% Cl	*p*-value	HR	95% Cl	*p*-value
Age	1.018	1.006–1.030	0.004	1.015	1.001–1.029	0.034
Temperature	1.234	1.034–1.471	0.020	1.291	1.070–1.559	0.008
DBP	0.987	0.976–0.999	0.034			
Albumin	0.956	0.923–0.990	0.011	0.962	0.927–0.999	0.406
LDH	1.000	1.000–1.001	< 0.001			
LAR	1.014	1.010–1.018	< 0.001	1.010	1.006–1.015	< 0.001
WBC	1.033	1.010–1.056	0.005			
Neutrophil	1.040	1.013–1.067	0.003	1.052	1.021–1.084	< 0.001
PCT	1.012	1.004–1.019	0.002			
ALT	1.001	1.000–1.001	0.019			
BUN	1.031	1.014–1.049	< 0.001			
Creatinine	1.002	1.001–1.003	< 0.001			
Uric acid	1.001	1.000–1.002	0.023			
AG	1.081	1.038–1.126	< 0.001			
SOFA	1.236	1.169–1.307	< 0.001	1.224	1.141–1.313	< 0.001
qSOFA	1.415	1.148–1.745	0.001			
CURB-65	1.518	1.277–1.805	< 0.001			
COVID-19	2.316	1.669–3.212	< 0.001	3.060	2.121–4.413	< 0.001
Sepsis	1.923	1.389–2.663	< 0.001	1.614	1.130–2.307	0.009

### 3.4 Non-linear relationship analysis

To further investigate whether there is a non-linear relationship between LAR and 28-day mortality, RCS method was used for visualization analysis, with the number of knots set to 4. After adjusting for covariates identified in the multivariate Cox analysis, the RCS analysis revealed a significant non-linear association between LAR and 28-day mortality (Figure 1C; *P* overall < 0.001, *P* non-linear < 0.001). This result suggests that as LAR levels increase, the risk of death rises in a non-linear manner, further emphasizing its potential role in clinical risk stratification.

### 3.5 Boruta algorithm analysis

**Figure 2:**
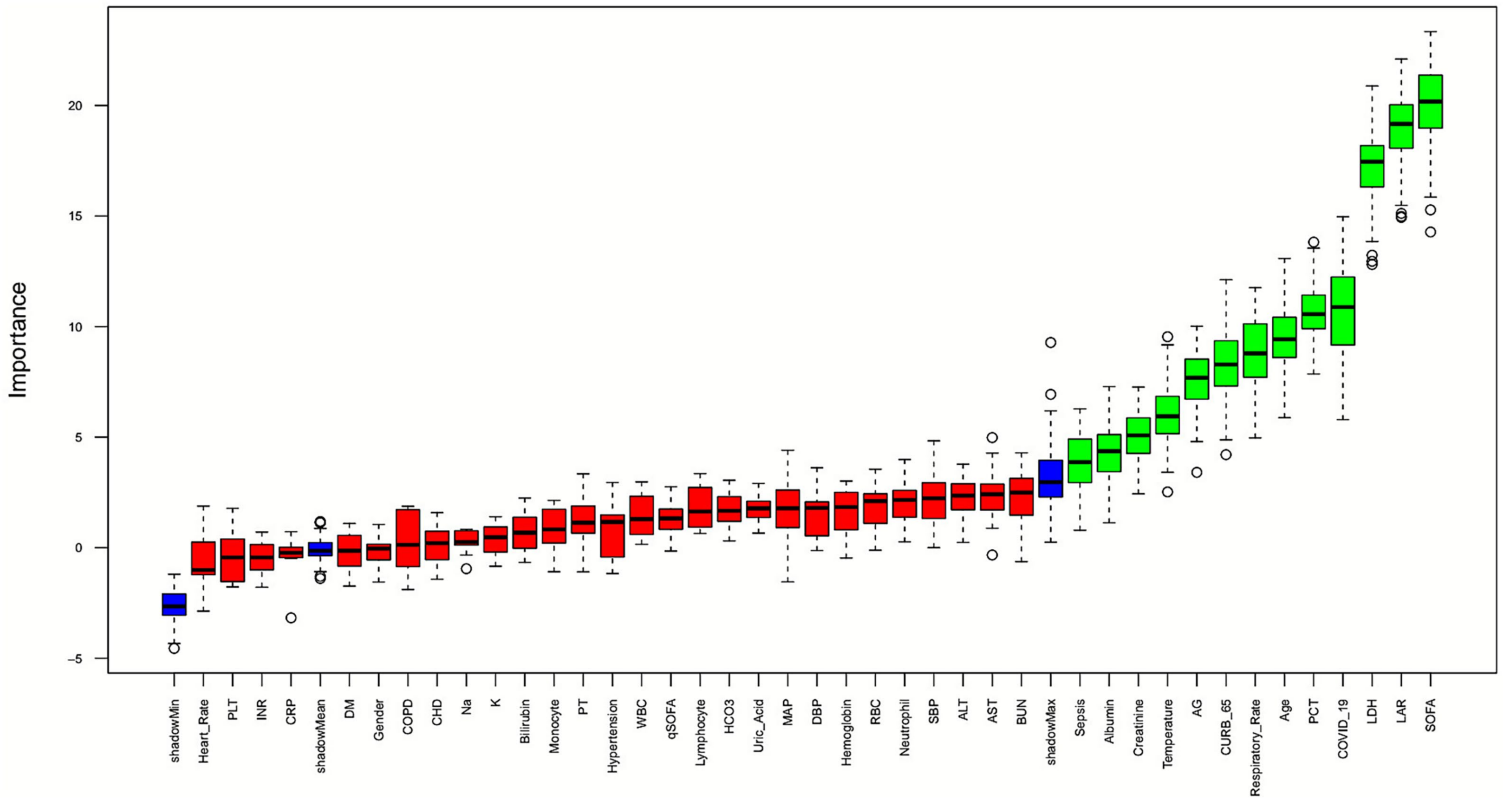
Boruta analysis. The Boruta algorithm is utilized for feature selection. The x-axis denotes the names of the variables, while the y-axis represents the Z scores of each variable. The box plot illustrates the distribution of Z scores for each variable during the model computation process.

To identify the key variables influencing 28-day mortality in severe pneumonia patients, the Boruta algorithm was used to rank the importance of all candidate features (Figure 2). In the output visualization, green represents variables identified as “important,” while red represents “unimportant” variables. The important variables included Age, Temperature, Albumin, LDH, LAR, PCT, Creatinine, AG, SOFA, CURB-65, and comorbidities such as COVID-19 and Sepsis. These results were highly consistent with the findings from the previous univariate Cox regression analysis, demonstrating the stability and reliability of the algorithm in feature selection. Since the SOFA score is a composite index comprising multiple indicators, it was not included in the model. Notably, LAR was evaluated as the second most important feature after the SOFA score, highlighting its critical role in predicting short-term mortality risk. Additionally, Respiratory Rate was also identified as an important variable.

### 3.6 Subgroup analysis and interaction testing

**Figure 3:**
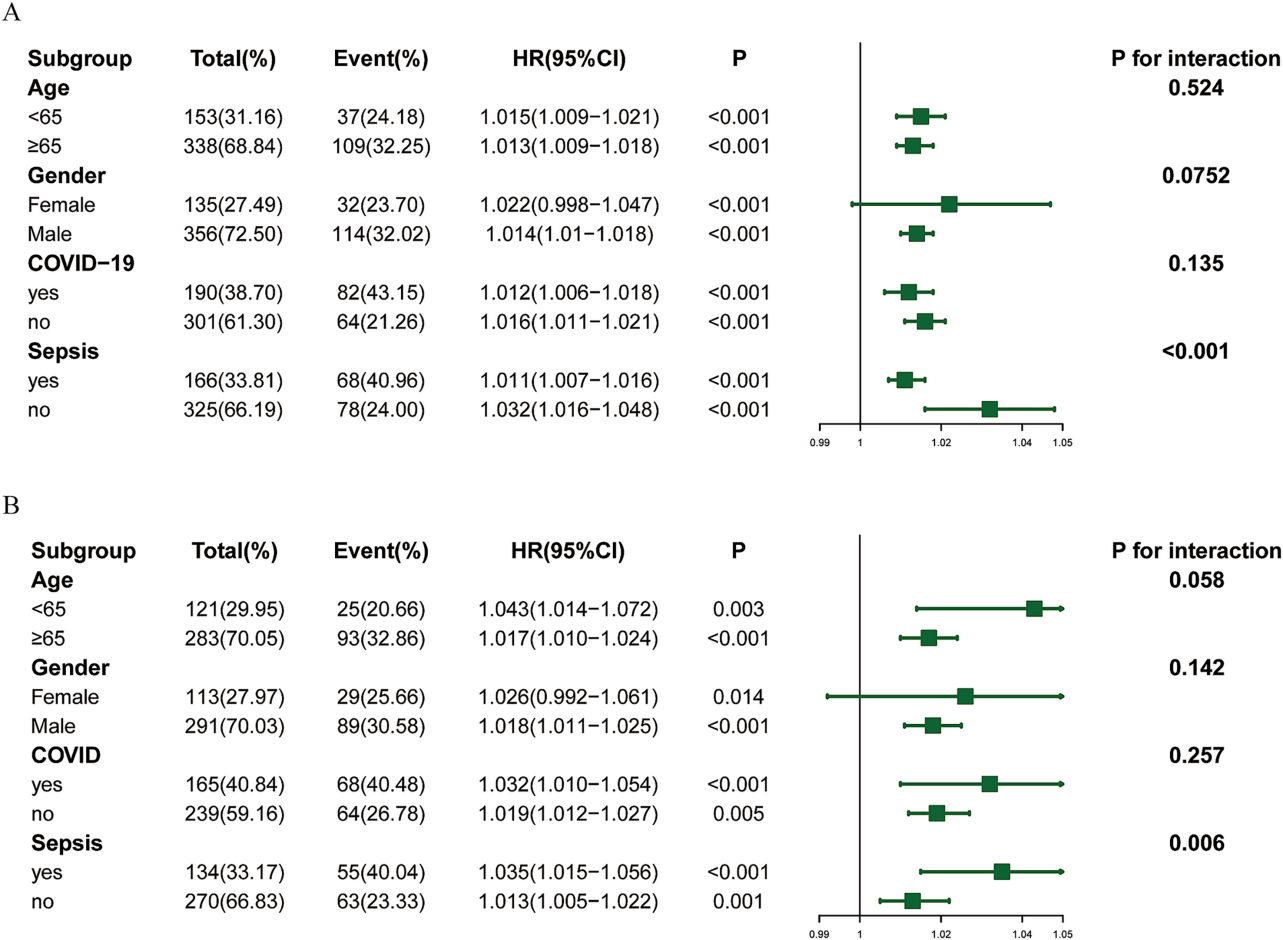
Subgroup analysis. **(A)** Subgroup analysis and forest plot of LAR and mortality in patients with severe pneumonia. **(B)** Subgroup sensitivity analysis after excluding data from the most recent year.

To further validate the stability and applicability of LAR in predicting 28-day mortality risk in severe pneumonia patients across different clinical contexts, we conducted subgroup analyses and interaction tests based on key demographic and clinical factors (Figure 3). Subgroup divisions were made according to the following variables: age (< 65 years vs. ≥ 65 years), gender (male vs. female), comorbidity with COVID-19, and comorbidity with Sepsis. In most subgroups, LAR showed a significant positive correlation with 28-day mortality risk (HR > 1, *p* < 0.05) (Figure 3A). To verify the robustness of the results, a sensitivity analysi**s** was performed. After excluding data from the most recent year (May 2024 to May 2025), the HRs and their trends across subgroups (Figure 3B) remained consistent with those obtained from the original analysis (Figure 3A), indicating good robustness of the findings.

Further interaction analysis revealed that the association between LAR and mortality risk significantly differed in patients with or without comorbid sepsis (*P* for interaction < 0.001). This suggests that sepsis may act as a moderating variable, influencing the predictive ability of LAR for mortality risk. In contrast, no significant interaction effects were observed for the other subgroup variables (age, gender, COVID-19) (*p* > 0.05). This phenomenon may reflect the following mechanism: in patients with comorbid sepsis, the baseline mortality risk is already significantly elevated and driven by multiple factors, which could overshadow the relative predictive contribution of LAR, one of the indicators. Additionally, the smaller sample sizes in some subgroups may affect the statistical significance of the interaction effects.

### 3.7 Construction and validation of the predictive model

In accordance with the study design, patients were randomly divided into training and validation sets in a 3:1 ratio, with the training set comprising 369 patients and the validation set comprising 122 patients. Regarding the 28-day mortality rate, 110 patients (29.8%) in the training set and 36 patients (29.5%) in the validation set died. No statistically significant differences were observed between the two groups in terms of clinical characteristics (*p* > 0.05) (Table 3).

**Table 3:** Characteristics of patient in training set and validation set.

Characteristics	Training *N* = 369	Validation *N* = 122	*p*-value
Age (years)	72 (60–80)	71 (60–80)	0.832
Temperature (°C)	36.7 (36.5–37.4)	36.7 (36.5–37)	0.135
Respiratory rate (bpm)	22 (20–25)	21 (20–25)	0.408
Albumin (mg/dL)	29.1 (26.3–33)	29.3 (26.1–32.6)	0.822
PCT (ng/ml)	0.56 (0.19–2.26)	0.66 (0.20–1.54)	0.931
AG (mmol/L)	17.5 (15.8–20.00)	17.15 (15.50–19.40)	0.335
Creatinine (mg/dL)	87 (62–134)	79.9 (62–141)	0.836
LDH (U/L)	361 (278.7–542)	371 (263–530)	0.818
LAR	12.63 (9.40–18.46)	12.93 (9.41–17.02)	0.924
COVID, *n* (%)	142 (38.5%)	48 (39.3%)	0.865
Sepsis, *n* (%)	124 (33.6%)	42 (34.4%)	0.868
Mortality, *n* (%)	110 (29.8%)	36 (29.5%)	0.950

**Figure 4:**
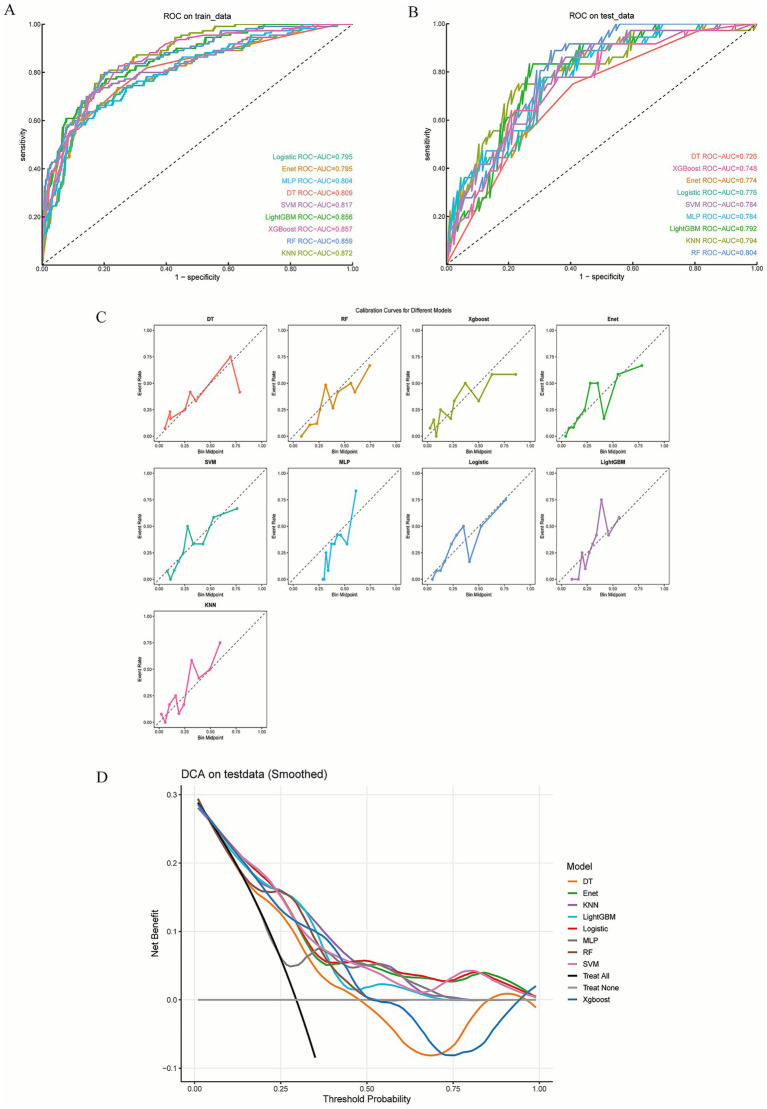
Performance comparison of Nine machine learning models. **(A)** ROC curve for the training set. **(B)** ROC curve for the validation set. **(C)** Calibration curve for multiple models in the validation set. **(D)** Decision curve for multiple models in the validation set.

Based on the important variables identified by the Boruta algorithm, nine commonly used machine learning models were constructed and trained to predict 28-day mortality risk in severe pneumonia. Bayesian Optimization was employed for hyperparameter tuning (Supplementary Table 1), and 10-fold cross-validation was used for internal validation, with all models demonstrating stable performance (Supplementary Table 3). Model performance was comprehensively evaluated in both the training and validation sets using AUC, accuracy, sensitivity, and specificity (Supplementary Table 2). The results showed that the RF model achieved the best performance, with an AUC of 0.804 in the validation set, outperforming other models (LightGBM: 0.792, KNN: 0.794, MLP: 0.784, SVM: 0.784, Logistic: 0.776, ENet: 0.774, XGBoost: 0.748, DT: 0.726) (Figures 4A,B). The learning curve (Supplementary Figure 1) demonstrated that the RF model exhibited gradual convergence between the training and validation sets without signs of overfitting or underfitting. Calibration analysis showed that all models had low Brier scores (0.009–0.035) and non-significant Hosmer–Lemeshow test results (*p* > 0.05), indicating good calibration. Among them, the RF, XGBoost and Logistic models were closest to the ideal diagonal line, showing the best calibration performance (Figure 4C; Supplementary Table 7). Decision curve analysis (Figure 4D) further revealed that RF, LightGBM, and Logistic achieved higher net benefits across a wide range of threshold probabilities, suggesting greater clinical applicability.

In summary, the RF model demonstrated superior predictive performance, stability, and clinical utility, and was therefore selected as the final predictive model.

### 3.8 Model interpretability analysis

**Figure 5:**
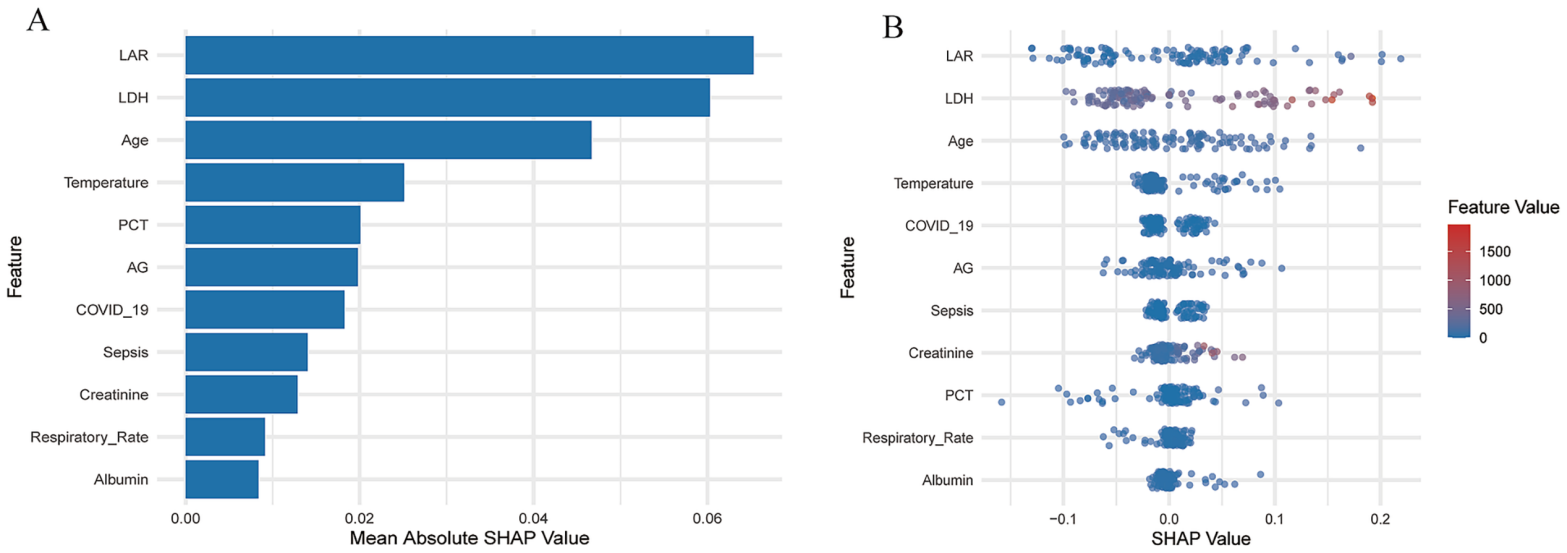
SHAP analysis of the RF model. **(A)** Feature importance ranking plot. **(B)** SHAP beeswarm plot.

Based on SHAP interpretability, the risk factors for 28-day mortality in severe pneumonia patients were analyzed using the best-performing model. Figure 5A displays the overall importance of the 11 feature variables included in the model. Each row represents a variable, and the x-axis indicates its corresponding SHAP value. Figure 5B shows the SHAP values for each sample, with each point representing a sample. The greater the number of points shown, the larger the variation of the feature across different samples. Points colored in red indicate higher values of that feature, while points in blue represent lower values. The SHAP analysis showed that LAR contributed the most to the model’s predictive performance, with the highest mean absolute SHAP value (mean |SHAP| = 0.068), which was significantly higher than those of other variables (PCT: 0.020, Albumin: 0.008, Age: 0.047) (Supplementary Table 6). Patients with higher LAR values generally exhibited a higher risk of death, indicating that LAR was the most influential feature affecting the model output and played an important role in predicting 28-day mortality risk.

## 4 Discussion

This study systematically evaluated the prognostic value of LAR in patients with severe pneumonia and emphasized its crucial role in predicting the 28-day mortality risk. Through multivariate Cox regression analysis, LAR was identified as an independent risk factor for 28-day mortality. Moreover, ROC analysis revealed that the AUC of LAR was superior to that of LDH, albumin, and commonly used clinical scoring systems (SOFA, qSOFA, and CURB-65), suggesting that LAR holds greater potential for short-term prognosis assessment. Feature selection using the Boruta algorithm highlighted LAR as one of the most significant variables influencing mortality risk. Subsequently, a 28-day mortality risk prediction model was constructed using nine machine learning algorithms, with the RF model demonstrating the best overall performance in both the training and validation sets. In the RF model, SHAP value analysis further confirmed that LAR was the most influential variable. Additionally, the RCS curve revealed an L-shaped non-linear relationship between LAR and 28-day mortality risk, indicating that the risk of death increases rapidly once LAR exceeds a certain threshold.

This study expands the application of the LAR to patients with severe pneumonia, a high-risk population. Existing studies have predominantly used LDH as a single biomarker to predict mortality associated with pneumonia (22–25). However, these studies failed to comprehensively reflect systemic changes such as inflammation, nutritional status, and capillary leakage. By incorporating serum albumin into the analysis, LAR provides a more holistic assessment of the patient’s metabolic reserve, liver function, and inflammatory burden. As a result, LAR holds significant advantages in the clinical evaluation of severe pneumonia. The current evidence further supports the prognostic value of LAR across various clinical settings. For instance, a retrospective cohort study by Xia et al. on 130 neonates with sepsis demonstrated that elevated LAR was associated with poor outcomes in neonatal sepsis (26). Moreover, as a biomarker, LAR outperforms traditional scoring systems (such as SAPSII and SOFA) and other biomarkers (including PNI, SIRI, and SII) in predicting 28-day ICU mortality and in-hospital mortality, and it can be used for mortality risk stratification in patients with malignancy-related sepsis (27). Research by Lee et al. (28) demonstrated that in patients with lower respiratory tract infections, the AUC of LAR significantly exceeded that of CURB-65 and PSI scores, confirming LAR as a reliable predictor of in-hospital mortality. In breast cancer patients, LAR was found to be negatively correlated with progression-free survival (PFS) and identified as an independent prognostic marker for breast cancer (29). These studies further validate the prognostic value of LAR in various disease contexts (30–32). The findings from this study confirm that LAR is an independent risk factor for 28-day mortality in patients with severe pneumonia, with a positive correlation to mortality risk. These results provide compelling evidence for the use of LAR as a tool for prognostic assessment in severe pneumonia.

The Boruta algorithm is a robust feature selection method that identifies the most critical variables for predictive tasks by comparing the importance of each feature with randomly shuffled “shadow features” (33). This algorithm retains variables that are more important than the shadow features while discarding those with minimal impact on predictive ability. In this study, the feature selection results from the Boruta algorithm demonstrated that the LAR plays a significant role in predicting the 28-day mortality risk in patients with severe pneumonia. This finding is highly consistent with the results of the Cox regression analysis, further validating the value of LAR as an important prognostic feature. Therefore, this study provides strong clinical evidence supporting LAR as a predictive indicator for 28-day mortality in patients with severe pneumonia.

In this study, the PCT levels among patients with severe pneumonia were generally moderate to low, possibly due to viral infections (such as COVID-19), prior antibiotic treatment, and early testing. Nevertheless, PCT remained significantly different between groups and was identified by the Boruta algorithm as an important predictive factor, indicating that it has certain prognostic value. The SOFA is currently the widely recognized standard for assessing prognosis and severity in critically ill patients (34), but its clinical application still has certain limitations. The SOFA primarily focuses on organ dysfunction and does not fully account for other crucial factors influencing the prognosis of critically ill patients, such as age, underlying comorbidities, and nutritional status. Therefore, in the predictive model constructed based on the LAR, this study incorporated multiple factors, including age, respiratory status, metabolic status, and nutritional condition, with the aim of providing a more comprehensive assessment of mortality risk in patients with severe pneumonia. To further enhance prediction accuracy, we employed multiple machine learning models, including DT, RF, and Xgboost, and performed both training and validation. The results showed that the RF model exhibited excellent performance in both the training and validation sets, with an accuracy rate of 74.59% in the test set. This high performance indicates that a multivariable machine learning approach can significantly improve the prediction of 28-day mortality in severe pneumonia patients and provide effective support for early prognosis assessment. This method can help clinicians identify patients at risk of deterioration, allowing for more proactive interventions such as optimizing hemodynamics, improving nutritional support, and close monitoring, potentially reducing mortality. Furthermore, Wu et al. compared traditional logistic regression with machine learning models in predicting mortality in adult sepsis patients. The results showed that the RF model outperformed both the logistic regression model and the SOFA and APACHE scoring systems in predicting sepsis mortality (35). Xiong et al. also confirmed that, compared to SVM and Logistic Regression, RF exhibited superior performance in identifying mortality risk in critically ill COVID-19 patients (36). These studies further validate the exceptional performance of the RF model in various prediction scenarios and highlight its significant potential in clinical monitoring and decision-making for interventions.

In the subgroup analysis, we explored the prognostic differences of the LAR across different groups. The results revealed that the association between LAR and mortality risk was more significant in non-septic patients, suggesting that septic status may substantially influence the role of LAR in prognostic assessment. Specifically, in non-septic patients, the HR for LAR was 1.032, significantly higher than the HR of 1.011 observed in septic patients. This indicates that LAR has a stronger independent predictive capacity for mortality in non-septic patients. This phenomenon may be closely related to the physiological condition of septic patients. In sepsis, due to the intense inflammatory response, widespread metabolic disturbances, and organ dysfunction (37), the prognostic reflection of LAR may be interfered with by other sensitive biomarkers, such as PCT (38), CRP (39, 40), SOFA and lactate levels (41), thereby diminishing its ability as a standalone prognostic indicator. Furthermore, LDH and albumin levels in septic patients are often influenced by various factors: LDH may be elevated due to extensive cellular damage, while albumin levels may rapidly decline due to acute-phase reactions and capillary leak syndrome, potentially leading to an increase in LAR. However, because septic patients often exhibit elevated levels of multiple inflammatory and injury biomarkers, the changes in LAR may lack specificity, which weakens its correlation with mortality risk. In contrast, in severe pneumonia patients without sepsis, elevated LAR is more likely to directly reflect the inflammatory damage and deterioration of nutritional status caused by the disease itself, thus providing stronger prognostic capacity. In conclusion, LAR demonstrates superior mortality prediction ability in non-septic severe pneumonia patients.

This study has several limitations. First, as a single-center retrospective study, the model’s generalizability may be limited. The patient cohort from one hospital may lack representativeness in age, comorbidities, and socioeconomic background, potentially introducing selection bias. Moreover, specific diagnostic workflows and regional pathogen patterns may restrict the model’s applicability in other clinical settings. At present, there is no internationally standardized method for measuring or calculating LAR. Differences in laboratory methodologies across hospitals may lead to variability in LAR values and optimal cutoff points, limiting its generalizability and comparability among different populations. Future large-scale prospective studies are needed to promote the standardization of LAR. A prospective, multicenter external validation study is planned to further evaluate the model’s robustness, generalizability, and potential for clinical implementation. Second, baseline data of excluded patients (e.g., those with malignancy or hepatic/renal insufficiency) were not collected, precluding comparison with the included cohort and potentially limiting external applicability. Future studies should include a broader population and perform subgroup or sensitivity analyses to verify model stability. Third, this study used the lactate-to-albumin ratio (LAR) within 24 h after admission to meet the need for early risk prediction. However, dynamic changes in LAR may more accurately reflect disease progression and treatment response. Due to the retrospective design, this study was unable to systematically analyze these trends. Future prospective studies monitoring longitudinal LAR trajectories and correlating them with key clinical events (e.g., vasopressor use, intubation, ICU transfer, and mortality) could further explore its potential as a predictive biomarker. In addition, the events-per-variable (EPV) in this study was approximately 11, which is below the ideal standard (≥20–50) and may affect model stability. We mitigated this issue through feature selection, regularization, and cross-validation; however, further validation in larger samples is warranted. Despite these limitations, this study still offers significant advantages. By combining traditional statistical methods with advanced machine learning techniques, this research has enhanced the robustness and predictive power of the results. This innovative approach has improved the reliability and comprehensiveness of LAR as a prognostic indicator, providing a solid foundation for future research and potential clinical applications.

## 5 Conclusion

This study identifies the LAR as a significant predictor of 28-day mortality in patients with severe pneumonia. Elevated LAR is closely associated with an increased risk of adverse outcomes. The machine learning model incorporating LAR demonstrated robust predictive performance for poor prognosis in this patient cohort. Despite certain limitations, this study provides solid evidence supporting the clinical application of LAR as a predictive indicator for mortality risk in severe pneumonia, with important implications for both clinical practice and future research.

## Data Availability

The raw data supporting the conclusions of this article will be made available by the authors, without undue reservation.

## Supplementary material

Supplementary Figure 1 RF_Learning_Curve.

## References

[1] Sligl WI Marrie TJ. Severe community-acquired pneumonia. Crit Care Clin. (2013) 29:563–601. doi: 10.1016/j.ccc.2013.03.009, 23830654 PMC7126707

[2] Qu J Zhang J Chen Y Huang Y Xie Y Zhou M . Aetiology of severe community acquired pneumonia in adults identified by combined detection methods: a multi-Centre prospective study in China. Emerg Microbes Infect. (2022) 11:556–66. doi: 10.1080/22221751.2022.2035194, 35081880 PMC8843176

[3] Garnacho-Montero J Barrero-García I Gómez-Prieto MG Martín-Loeches I. Severe community-acquired pneumonia: current management and future therapeutic alternatives. Expert Rev Anti-Infect Ther. (2018) 16:667–77. doi: 10.1080/14787210.2018.1512403, 30118377

[4] Nair GB Niederman MS. Year in review 2013: critical care--respiratory infections. Crit Care. (2014) 18:572. doi: 10.1186/s13054-014-0572-3, 25672674 PMC4330923

[5] Hicks KG Cluntun AA Schubert HL Hackett SR Berg JA Leonard PG . Protein-metabolite interactomics of carbohydrate metabolism reveal regulation of lactate dehydrogenase. Science. (2023) 379:996–1003. doi: 10.1126/science.abm3452, 36893255 PMC10262665

[6] Liang W Liang H Ou L Chen B Chen A Li C . Development and validation of a clinical risk score to predict the occurrence of critical illness in hospitalized patients with COVID-19. JAMA Intern Med. (2020) 180:1081–9. doi: 10.1001/jamainternmed.2020.2033, 32396163 PMC7218676

[7] Meng X Zhu Y Yang W Zhang J Jin W Tian R . HIF-1α promotes virus replication and cytokine storm in H1N1 virus-induced severe pneumonia through cellular metabolic reprogramming. Virol Sin. (2024) 39:81–96. doi: 10.1016/j.virs.2023.11.010, 38042371 PMC10877445

[8] Martinez Mesa A Cabrera César E Martín-Montañez E Sanchez Alvarez E Lopez PM Romero-Zerbo Y . Acute lung injury biomarkers in the prediction of COVID-19 severity: Total thiol, ferritin and lactate dehydrogenase. Antioxidants. (2021) 10. doi: 10.3390/antiox10081221, 34439469 PMC8388961

[9] Liu Z Liu F Liu C Chen X. Association between lactate dehydrogenase and 30-day mortality in patients with sepsis: a retrospective cohort study. Clin Lab. (2023) 69. doi: 10.7754/Clin.Lab.2022.220915 37307113

[10] Su Y Ju MJ Ma JF Tu GW He HY Gu ZY . Lactate dehydrogenase as a prognostic marker of renal transplant recipients with severe community-acquired pneumonia: a 10-year retrospective study. Ann Transl Med. (2019) 7:660. doi: 10.21037/atm.2019.10.75, 31930061 PMC6944597

[11] Tessari P. Protein metabolism in liver cirrhosis: from albumin to muscle myofibrils. Curr Opin Clin Nutr Metab Care. (2003) 6:79–85. doi: 10.1097/00075197-200301000-00012, 12496684

[12] Wu MA Fossali T Pandolfi L Carsana L Ottolina D Frangipane V . Hypoalbuminemia in COVID-19: assessing the hypothesis for underlying pulmonary capillary leakage. J Intern Med. (2021) 289:861–72. doi: 10.1111/joim.13208, 33411411

[13] Fanali G di Masi A Trezza V Marino M Fasano M Ascenzi P. Human serum albumin: from bench to bedside. Mol Asp Med. (2012) 33:209–90. doi: 10.1016/j.mam.2011.12.002, 22230555

[14] Kumar M Jain K Chauhan R Meena SC Luthra A Thakur H . Hypoalbuminemia: incidence and its impact on acute respiratory distress syndrome and 28-day outcome in trauma patients. Eur J Trauma Emerg Surg. (2023) 49:2305–14. doi: 10.1007/s00068-023-02318-5, 37402792

[15] Viana-Llamas MC Arroyo-Espliguero R Silva-Obregón JA Uribe-Heredia G Núñez-Gil I García-Magallón B . Hypoalbuminemia on admission in COVID-19 infection: an early predictor of mortality and adverse events. A retrospective observational study. Med Clin (Barc). (2021) 156:428–36. doi: 10.1016/j.medcli.2020.12.018, 33627230 PMC7843155

[16] Turcato G Zaboli A Sibilio S Rella E Bonora A Brigo F. Albumin as a prognostic marker of 30-day mortality in septic patients admitted to the emergency department. Intern Emerg Med. (2023) 18:2407–17. doi: 10.1007/s11739-023-03387-5, 37563529

[17] Jeon SY Ryu S Oh SK Park JS You YH Jeong WJ . Lactate dehydrogenase to albumin ratio as a prognostic factor for patients with severe infection requiring intensive care. Medicine. (2021) 100:e27538. doi: 10.1097/MD.0000000000027538, 34731152 PMC8519202

[18] Xiao X Wu JJ Liu Y Suo Z Zhang H Xu HB. Increased lactate dehydrogenase to albumin ratio is associated with short-term mortality in septic ICU patients: a retrospective cohort study. Medicine. (2024) 103:e40854. doi: 10.1097/MD.0000000000040854, 39969341 PMC11688072

[19] Sipahioglu H Onuk S. Lactate dehydrogenase/albumin ratio as a prognostic factor in severe acute respiratory distress syndrome cases associated with COVID-19. Medicine. (2022) 101:e30759. doi: 10.1097/MD.0000000000030759, 36197158 PMC9508955

[20] Mandell LA Wunderink RG Anzueto A Bartlett JG Campbell GD Dean NC . Infectious Diseases Society of America/American Thoracic Society consensus guidelines on the management of community-acquired pneumonia in adults. Clin Infect Dis. (2007) 44:S27–72. doi: 10.1086/511159, 17278083 PMC7107997

[21] Luckhurst CM Wiberg HM Brown RL Bruch SW Chandler NM Danielson PD . Pediatric cervical spine injury following blunt trauma in children younger than 3 years: the PEDSPINE II study. JAMA Surg. (2023) 158:1126–32. doi: 10.1001/jamasurg.2023.4213, 37703025 PMC10500431

[22] Wang J Wang R Zhou Y Ma Y Xiong C. The relationship between lactate dehydrogenase and Apolipoprotein A1 levels in patients with severe pneumonia. J Med Biochem. (2024) 43:290–8. doi: 10.5937/jomb0-45782, 38699695 PMC11062332

[23] Cheng X Liu L Tian Y Lin Y. Serum lactate dehydrogenase as a prognostic marker for 90-day mortality in connective tissue disease patients receiving glucocorticoids and hospitalized with pneumonia: a cohort study. Sci Rep. (2025) 15:16806. doi: 10.1038/s41598-025-01721-9, 40369099 PMC12078684

[24] Koh MCY Ngiam JN Tambyah PA Lum LH. Elevated serum lactate dehydrogenase aids prediction of mortality in *pneumocystis jirovecii* pneumonia without underlying human immunodeficiency virus infection - derivation of a clinical risk score. J Infect Public Health. (2024) 17:102439. doi: 10.1016/j.jiph.2024.04.023, 38820900

[25] Qian X Sheng Y Jiang Y Xu Y. Association between lactate dehydrogenase and ventilator-associated pneumonia risk: an analysis of the MIMIC database 2001-2019. BMC Pulm Med. (2024) 24:273. doi: 10.1186/s12890-024-03084-9, 38844914 PMC11157856

[26] Xia X Qiu S Cheng X Xie M Zhou J. Lactate dehydrogenase to albumin ratio as an independent factor for 28-day mortality of neonatal sepsis. Sci Rep. (2025) 15:15158. doi: 10.1038/s41598-025-89108-8, 40307261 PMC12043797

[27] Shen Y Lin K Yang L Zheng P Zhang W Weng J . Association between the lactate dehydrogenase-to-albumin ratio and 28-day mortality in septic patients with malignancies: analysis of the MIMIC-IV database. BMC Cancer. (2025) 25:637. doi: 10.1186/s12885-025-14013-2, 40200294 PMC11980078

[28] Lee BK Ryu S Oh SK Ahn HJ Jeon SY Jeong WJ . Lactate dehydrogenase to albumin ratio as a prognostic factor in lower respiratory tract infection patients. Am J Emerg Med. (2022) 52:54–8. doi: 10.1016/j.ajem.2021.11.028, 34864628

[29] He J Tong L Wu P Wu Y Shi W Chen L. Prognostic significance of preoperative lactate dehydrogenase to albumin ratio in breast Cancer: a retrospective study. Int J Gen Med. (2023) 16:507–14. doi: 10.2147/IJGM.S396871, 36789133 PMC9922482

[30] Li N Feng Y Chen X Zou LQ. The prognostic value of lactate dehydrogenase/albumin ratio in extranodal natural killer/T cell lymphoma. BMC Cancer. (2025) 25:1176. doi: 10.1186/s12885-025-14393-5, 40665221 PMC12261561

[31] Zhang R Mu Y Zhang M Wu H Xu Y Zhao C . Lactate dehydrogenase/albumin to urea ratio: a novel prognostic Indicator for adverse outcomes in patients with severe fever with thrombocytopenia syndrome. J Med Virol. (2025) 97:e70428. doi: 10.1002/jmv.70428, 40474649 PMC12142283

[32] Hu J Zhou Y. The association between lactate dehydrogenase to serum albumin ratio and in-hospital mortality in patients with pulmonary embolism: a retrospective analysis of the MIMIC-IV database. Front Cardiovasc Med. (2024) 11:1398614. doi: 10.3389/fcvm.2024.1398614, 38962086 PMC11220285

[33] Yan F Chen X Quan X Wang L Wei X Zhu J. Association between the stress hyperglycemia ratio and 28-day all-cause mortality in critically ill patients with sepsis: a retrospective cohort study and predictive model establishment based on machine learning. Cardiovasc Diabetol. (2024) 23:163. doi: 10.1186/s12933-024-02265-4, 38725059 PMC11084034

[34] Asai N Watanabe H Shiota A Kato H Sakanashi D Hagihara M . Efficacy and accuracy of qSOFA and SOFA scores as prognostic tools for community-acquired and healthcare-associated pneumonia. Int J Infect Dis. (2019) 84:89–96. doi: 10.1016/j.ijid.2019.04.020, 31028877

[35] Wu H Liao B Ji T Ma K Luo Y Zhang S. Comparison between traditional logistic regression and machine learning for predicting mortality in adult sepsis patients. Front Med. (2024) 11:1496869. doi: 10.3389/fmed.2024.1496869, 39835102 PMC11743956

[36] Xiong Y Ma Y Ruan L Li D Lu C Huang L. Comparing different machine learning techniques for predicting COVID-19 severity. Infect Dis Poverty. (2022) 11:19. doi: 10.1186/s40249-022-00946-4, 35177120 PMC8851750

[37] Li B Lin W Hu R Bai S Ruan Y Fan Y . Crosstalk between lung and extrapulmonary organs in sepsis-related acute lung injury/acute respiratory distress syndrome. Ann Intensive Care. (2025) 15:97. doi: 10.1186/s13613-025-01513-4, 40658295 PMC12259525

[38] Wang D Wang X Mu J Kuang Z Zhang J Lu X . Prognostic indicators and outcome in patients with acute liver failure, sepsis and with and without shock: a retrospective cohort study. Ann Med. (2025) 57:2438833. doi: 10.1080/07853890.2024.2438833, 39661398 PMC11636143

[39] Chuang CL Yeh HT Niu KY Chen CB Seak CJ Yen CC. Diagnostic performances of procalcitonin and C-reactive protein for sepsis: a systematic review and meta-analysis. Eur J Emerg Med. (2025) 32:248–58. doi: 10.1097/MEJ.0000000000001235, 40214293

[40] Benhamou J Nieves-Ortega R Nickel CH Lampart A Kuster T Balestra GM . Human neutrophil lipocalin, procalcitonin, c-reactive protein, and leucocyte count for prediction of bacterial sepsis in emergency department patients. Scand J Trauma Resusc Emerg Med. (2025) 33:112. doi: 10.1186/s13049-025-01429-9, 40598497 PMC12210756

[41] Acharya CP Yadav A Pokhrel S Bastola S Jha S. Prognostic significance of lactate/albumin ratio in respiratory failure and sepsis. Ann Med. (2025) 57:2482024. doi: 10.1080/07853890.2025.2482024, 40130722 PMC11938314

