# Supplementary figures and images for: Establishment and validation of a 28-day mortality prediction model based on the lactate dehydrogenase/albumin ratio in patients with severe pneumonia
Source: Front Med (Lausanne). 2026 Jan 21;12:1696945. doi: 10.3389/fmed.2025.1696945 (PMC12869708; doi:10.3389/fmed.2025.1696945)

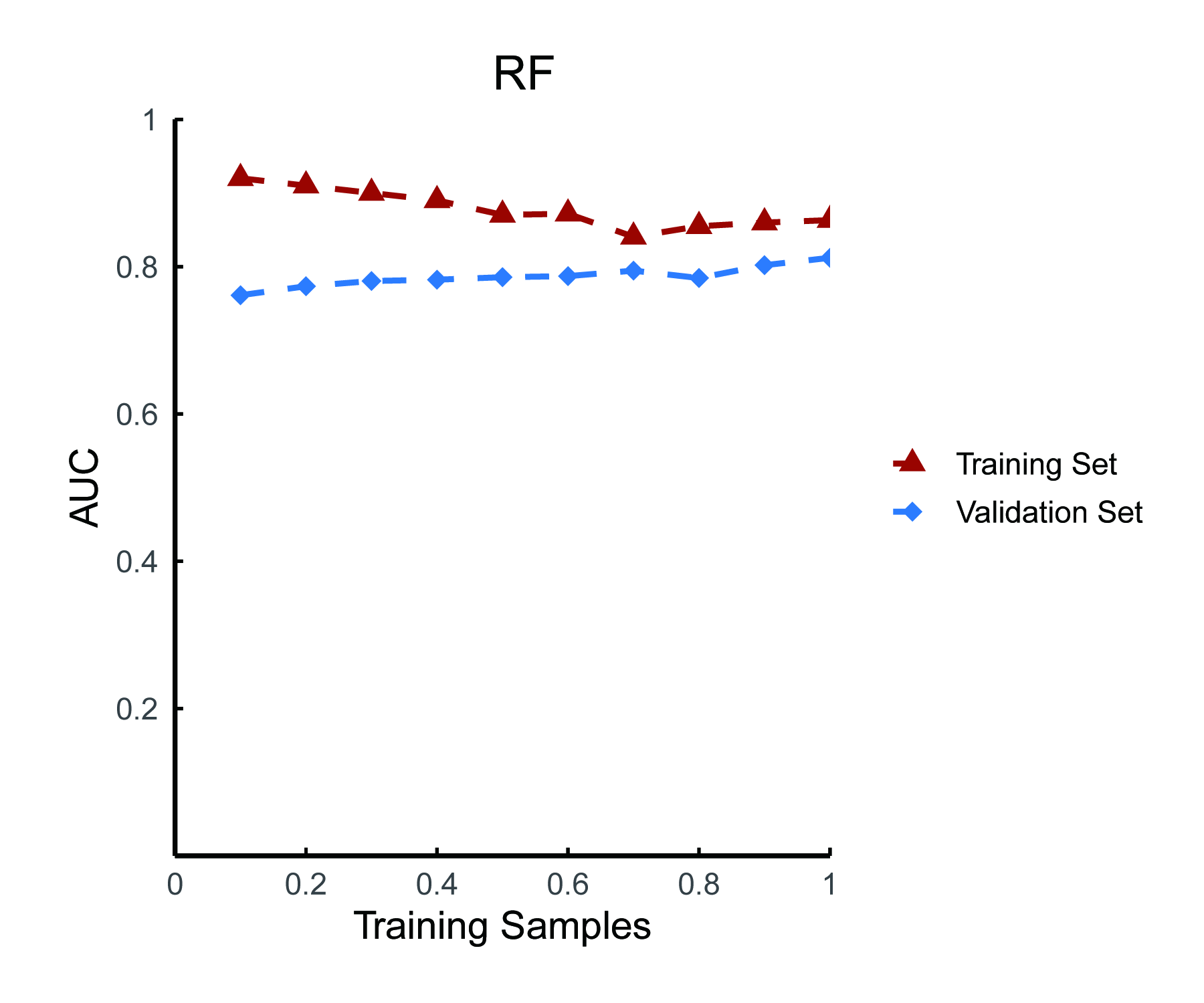

Supplement: Supplementary file 1 [file Image_1.tif]
