# Supplementary material for: Establishment and validation of a 28-day mortality prediction model based on the lactate dehydrogenase/albumin ratio in patients with severe pneumonia
Source: Front Med (Lausanne). 2026 Jan 21;12:1696945. doi: 10.3389/fmed.2025.1696945 (PMC12869708; doi:10.3389/fmed.2025.1696945)
Supplement: Supplementary file 2 [file Table_1.DOCX]

**Supplementary Table 1. Hyperparameter Configuration of Machine Learning Models**

| **Model** | **Hyperparameters** | **Hyperparameter Selection** |
| --- | --- | --- |
| Decision Tree | min_n | 23 |
|  | Tree depth | 6 |
|  | Cost_complexity | 0.012 |
| Random Forest | mtry | 1 |
|  | Trees | 622 |
|  | min_n | 120 |
| Xgboost | mtry | 11 |
|  | min_n | 6 |
|  | Tree depth | 5 |
|  | Learn rate | 1.024 |
|  | Loss reduction | 9.995 |
|  | Sample size | 0.923 |
| Enet | mixture | 0.026 |
|  | penalty | 0.003 |
| SVM | cost | 57025.211 |
|  | Rbf sigma | 0.0001 |
| MLP | Hidden units | 14 |
|  | penalty | 0.094 |
|  | epochs | 50 |
| Logistic Regression | penalty | 0.0001 |
| Light GBM | Tree depth | 2 |
|  | Trees | 1858 |
|  | learn_rate | 0.001 |
|  | mtry | 4 |
|  | min_n | 26 |
|  | loss_reduction | 2 |
| KNN | neighbors | 30 |

**Supplementary Table 2. Performance Metrics of the Nine Machine Learning Models**

| Model | | Accuracy | Sensitivity | Specificity | PPV | NPV | F1-Score | Roc_auc |
| --- | --- | --- | --- | --- | --- | --- | --- | --- |
| Decision Tree | Train | 0.710 | 0.664 | 0.818 | 0.896 | 0.508 | 0.763 | 0.809 |
|  | Test | 0.639 | 0.593 | 0.750 | 0.850 | 0.435 | 0.699 | 0.726 |
| Random Forest | Train | 0.783 | 0.800 | 0.776 | 0.703 | 0.901 | 0.788 | 0.860 |
|  | Test | 0.713 | 0.722 | 0.709 | 0.630 | 0.859 | 0.738 | 0.804 |
| Xgboost | Train | 0.775 | 0.827 | 0.753 | 0.587 | 0.911 | 0.687 | 0.857 |
|  | Test | 0.746 | 0.639 | 0.791 | 0.561 | 0.840 | 0.597 | 0.748 |
| Enet | Train | 0.751 | 0.736 | 0.757 | 0.563 | 0.871 | 0.638 | 0.795 |
|  | Test | 0.713 | 0.778 | 0.686 | 0.509 | 0.881 | 0.615 | 0.774 |
| SVM | Train | 0.810 | 0.700 | 0.857 | 0.675 | 0.871 | 0.688 | 0.817 |
|  | Test | 0.697 | 0.861 | 0.628 | 0.492 | 0.915 | 0.626 | 0.784 |
| MLP | Train | 0.772 | 0.645 | 0.826 | 0.612 | 0.846 | 0.628 | 0.804 |
|  | Test | 0.721 | 0.778 | 0.698 | 0.519 | 0.882 | 0.622 | 0.784 |
| Logistic Regression | Train | 0.794 | 0.627 | 0.865 | 0.663 | 0.845 | 0.645 | 0.795 |
|  | Test | 0.672 | 0.889 | 0.581 | 0.471 | 0.926 | 0.61 | 0.776 |
| LightGBM | train | 0.789 | 0.795 | 0.773 | 0.892 | 0.616 | 0.841 | 0.856 |
|  | test | 0.730 | 0.756 | 0.667 | 0.844 | 0.533 | 0.798 | 0.793 |
| KNN | Train | 0.805 | 0.791 | 0.811 | 0.640 | 0.901 | 0.707 | 0.872 |
|  | Test | 0.762 | 0.750 | 0.767 | 0.574 | 0.880 | 0.651 | 0.794 |

**Supplementary Table 3. Results of ten-fold cross-validation for nine machine learning models**

| Model | Accuracy (±SD) | Precision (±SD) | Recall (±SD) | F1-score (±SD) | AUC (±SD) |
| --- | --- | --- | --- | --- | --- |
| Decision Tree | 0.696 ± 0.078 | 0.754 ± 0.047 | 0.843 ± 0.112 | 0.793 ± 0.065 | 0.739 ± 0.091 |
| Random Forest | 0.748 ± 0.070 | 0.764 ± 0.082 | 0.934 ± 0.061 | 0.836 ± 0.051 | 0.762 ± 0.069 |
| XGBoost | 0.694 ± 0.079 | 0.762 ± 0.072 | 0.817 ± 0.130 | 0.787 ± 0.079 | 0.682 ± 0.095 |
| Elastic Net | 0.755 ± 0.048 | 0.776 ± 0.032 | 0.918 ± 0.064 | 0.839 ± 0.034 | 0.750 ± 0.068 |
| SVM | 0.705 ± 0.039 | 0.716 ± 0.034 | 0.970 ± 0.076 | 0.821 ± 0.032 | 0.741 ± 0.071 |
| MLP | 0.735 ± 0.046 | 0.765 ± 0.038 | 0.905 ± 0.072 | 0.827 ± 0.034 | 0.739 ± 0.063 |
| Logistic Regression | 0.759 ± 0.049 | 0.774 ± 0.036 | 0.931 ± 0.063 | 0.844 ± 0.034 | 0.768 ± 0.102 |
| Naive Bayes | 0.741 ± 0.056 | 0.766 ± 0.049 | 0.918 ± 0.086 | 0.832 ± 0.040 | 0.747 ± 0.083 |
| KNN | 0.708 ± 0.060 | 0.743 ± 0.034 | 0.894 ± 0.075 | 0.810 ± 0.044 | 0.711 ± 0.107 |

**Supplementary Table 4. Multicollinearity test of candidate variables (Variance Inflation Factor, VIF)**

| Variable | VIF | Collinearity_Level |
| --- | --- | --- |
| Age | 1.547 | Low |
| Temperature | 1.036 | Low |
| DBP | 1.210 | Low |
| Albumin | 2.102 | Low |
| LDH | 59.380 | High |
| LAR | 61.380 | High |
| WBC | 17.852 | High |
| Neutrophil | 18.335 | High |
| PCT | 1.252 | Low |
| ALT | 1.086 | Low |
| BUN | 2.892 | Low |
| Creatinine | 2.271 | Low |
| Uric_Acid | 2.123 | Low |
| AG | 1.530 | Low |
| SOFA | 1.425 | Low |
| qSOFA | 1.537 | Low |
| CURB_65 | 2.074 | Low |
| COVID | 1.163 | Low |
| Sepsis | 1.189 | Low |

**Supplementary Table 5. Multicollinearity test of candidate variables after removing LDH and WBC (Variance Inflation Factor, VIF)**

| Variable | VIF | Collinearity_Level |
| --- | --- | --- |
| Age | 1.508 | Low |
| Temperature | 1.036 | Low |
| DBP | 1.209 | Low |
| Albumin | 1.094 | Low |
| LAR | 1.140 | Low |
| Neutrophil | 1.096 | Low |
| PCT | 1.246 | Low |
| ALT | 1.082 | Low |
| BUN | 2.873 | Low |
| Creatinine | 2.268 | Low |
| Uric_Acid | 2.102 | Low |
| AG | 1.528 | Low |
| SOFA | 1.416 | Low |
| qSOFA | 1.533 | Low |
| CURB_65 | 2.064 | Low |
| COVID | 1.158 | Low |
| Sepsis | 1.183 | Low |

**Supplementary Table 6. Quantitative comparison of SHAP values between LAR and other key predictors**

| Variable | Mean_abs_shap | Mean_LAR | Wilcoxon_Stat | P_Value |
| --- | --- | --- | --- | --- |
| AG | 0.020 | 0.068 | 6820 | < 0.001 |
| Age | 0.047 | 0.068 | 4712 | 0.007 |
| Albumin | 0.008 | 0.068 | 7280 | < 0.001 |
| COVID_19 | 0.018 | 0.068 | 7033 | < 0.001 |
| Creatinine | 0.013 | 0.068 | 7318 | < 0.001 |
| PCT | 0.020 | 0.068 | 6629 | < 0.001 |
| Respiratory_Rate | 0.009 | 0.068 | 7315 | < 0.001 |
| Sepsis | 0.014 | 0.068 | 7180 | < 0.001 |
| Temperature | 0.025 | 0.068 | 6524 | < 0.001 |

**Supplementary Table 7. Quantitative assessment of calibration performance among nine machine learning models**

| Model | Brier_Score | HL_p_value |
| --- | --- | --- |
| SVM | 0.009 | 0.630 |
| Random Forest | 0.011 | 0.559 |
| Logistic | 0.011 | 0.565 |
| KNN | 0.013 | 0.236 |
| Elastic Net | 0.015 | 0.360 |
| XGBoost | 0.016 | 0.087 |
| Decision Tree | 0.018 | 0.086 |
| LightGBM | 0.020 | 0.132 |
| MLP | 0.035 | 0.063 |
